# Prospective clinical surveillance for severe acute respiratory illness and COVID-19 vaccine effectiveness in Kenyan hospitals during the COVID-19 pandemic

**DOI:** 10.1186/s12879-024-10140-6

**Published:** 2024-11-05

**Authors:** Ruth Khadembu Lucinde, Henry Gathuri, Lynda Isaaka, Morris Ogero, Livingstone Mumelo, Dennis Kimego, George Mbevi, Conrad Wanyama, Edwin Onyango Otieno, Stella Mwakio, Metrine Saisi, Elizabeth Isinde, Irene Njeri Oginga, Alvin Wachira, Evans Manuthu, Hazel Kariuki, Jared Nyikuli, Cyprian Wekesa, Amos Otedo, Hannah Bosire, Steve Biko Okoth, Winston Ongalo, David Mukabi, Wilber Lusamba, Beatrice Muthui, Isaac Adembesa, Caroline Mithi, Mohammed Sood, Nadia Ahmed, Bernard Gituma, Matiko Giabe, Charles Omondi, Rashid Aman, Patrick Amoth, Kadondi Kasera, Fred Were, Wangari Nganga, James A Berkley, Benjamin Tsofa, Jospeh Mwangangi, Philip Bejon, Edwine Barasa, Mike English, John Athony Gerard Scott, Samuel Akech, Eunice Wangeci Kagucia, Ambrose Agweyu, Anthony Oliwa Etyang

**Affiliations:** 1 KEMRI-Wellcome Trust Research Programme (KWTRP), Kilifi, Kenya; 2 Kiambu Level Five Hospital, Kiambu, Kenya; 3 Machakos Level Five Hospital, Machakos, Kenya; 4 Kitale County Referral Hospital, Kitale, Kenya; 5 Naivasha Level Five Hospital, Naivasha, Kenya; 6 Bungoma County Referral Hospital, Bungoma, Kenya; 7 Kisumu County Hospital, Kisumu, Kenya; 8 Kakamega County General Hospital, Kakamega, Kenya; 9 Busia County Referral Hospital, Busia, Kenya; 10 Mama Lucy Kibaki Hospital, Nairobi, Kenya; 11 Kenyatta University Teaching and Referral Hospital, Nairobi, Kenya; 12 Coast General Teaching and Referral Hospital, Mombasa, Kenya; 13 Kilifi County Hospital, Kilifi, Kenya; 14 Mbagathi County Hospital, Nairobi, Kenya; 15 Kisii Teaching and Referral Hospital, Kisii, Kenya; 16 Jaramogi Oginga Odinga Teaching and Referral Hospital, Kisumu, Kenya; 17 Ministry of Health, Government of Kenya, Nairobi, Kenya; 18 Kenya Paediatric Research Consortium, Nairobi, Kenya; 19 Presidential Policy & Strategy Unit, The Presidency, Government of Kenya, Nairobi, Kenya; 20 Centre for Tropical Medicine & Global Health, Nuffield Department of Medicine, University of Oxford, Oxford, United Kingdom; 21 London School of Hygiene and Tropical Medicine, London, United Kingdom

**Keywords:** Hospital surveillance, COVID-19, Severe acute respiratory illness, Vaccine effectiveness

## Abstract

**Background:**

There are limited data from sub-Saharan Africa describing the demographic characteristics, clinical features and outcome of patients admitted to public hospitals with severe acute respiratory infections during the COVID-19 pandemic.

**Methods:**

We conducted a prospective longitudinal hospital-based sentinel surveillance between May 2020 and December 2022 at 16 public hospitals in Kenya. All patients aged above 18 years admitted to adult medical wards in the participating hospitals were included. We collected data on demographic and clinical characteristics, SARS-CoV-2 infection and COVID-19 vaccination status and, admission episode outcomes. We determined COVID-19 vaccine effectiveness (VE) against admission with SARS-CoV-2 positive severe acute respiratory illness (SARI) (i.e., COVID-19) and progression to inpatient mortality among patients admitted with SARI, using a test-negative case control design.

**Results:**

Of the 52,636 patients included in the study, 17,950 (34.1%) were admitted with SARI. The median age was 50 years. Patients were equally distributed across sexes. Pneumonia was the most common diagnosis at discharge. Hypertension, Human Immunodeficiency Virus (HIV) infection and Diabetes Mellitus were the most common chronic comorbidities. SARS-CoV-2 test results were positive in 2,364 (27.9%) of the 8,471 patients that underwent testing. After adjusting for age, sex and presence of a chronic comorbidity, SARI patients were more likely to progress to inpatient mortality compared to non-SARI patients regardless of their SARS-CoV-2 infection status (adjusted odds ratio (aOR) for SARI and SARS-CoV-2 negative patients 1.22, 95% CI 1.10–1.37; and aOR for SARI and SARS-CoV-2 positive patients 1.32, 95% CI 1.24–1.40). After adjusting for age, sex and presence of a chronic comorbidity, COVID-19 VE against progression to inpatient mortality following admission with SARI for those with a confirmed vaccination status was 0.59 (95% CI 0.27–0.77).

**Conclusion:**

We have provided a comprehensive description of the demographic and clinical pattern of admissions with SARI in Kenyan hospitals during the COVID-19 pandemic period as well as the COVID-19 VE for these patients. These data were useful in providing situational awareness during the first three years of the pandemic in Kenya and informing national response measures.

**Supplementary Information:**

The online version contains supplementary material available at 10.1186/s12879-024-10140-6.

## Background

Unexpected disease outbreaks such as the COVID-19 pandemic pose unique challenges in characterising the clinical pattern of disease due to their novelty as well as the evolving pattern of clinical manifestation based on pathogen strains [1]. By 5th May 2023 when the COVID-19 pandemic was declared over by the World Health Organisation (WHO), Kenya had reported more than 340,000 confirmed cases and over 5,000 deaths due to COVID-19 against a global tally of over 770 million cases and almost 7 million deaths [2]. Three years into the pandemic, the pattern of disease among patients admitted to hospital during the period is still being described with key differences in severity and outcome by specific groups such as age, chronic comorbidities or ethnic groups being apparent [3–7]. However, data on these patterns in Kenyan and other sub-Saharan populations is limited despite indications that the clinical pattern of illness in these settings was significantly different to that in high-income countries [8].

Globally, hospital-based surveillance systems have been key in generating timely data on the evolving clinical pattern of COVID-19 and informing clinical practice [4, 5, 9–12]. These systems have been key in evaluating trends in mortality, admissions and disease burden as well as evaluating vaccine effectiveness against severe disease and mortality [9].

Kenya’s national COVID-19 vaccination programme begun on 5th March 2021. As at December 2023, over 23 million vaccine doses of six licensed vaccine products (Moderna, Pfizer/BioNTech, Gamaleya, Janssen(Johnson & Johnson), Oxford/AstraZeneca and Sinopharm) [13] had been delivered across the country [2]. However, vaccine coverage remains low at about 36% among adults [2, 14] while no local data on the effectiveness of these vaccines in the Kenyan population exist.

Working with Ministry of Health (MOH) officials in Kenya, we developed a hospital-based surveillance system using existing infrastructure from a clinical information network (CIN) [15–17] to conduct surveillance for admissions with severe acute respiratory illness (SARI) and COVID-19 in Kenya. Additionally, we used this platform to evaluate the effectiveness of licensed COVID-19 vaccines against admission with severe acute respiratory illness in Kenya.

This study describes the characteristics of adults admitted to the medical wards of 16 public hospitals with severe acute respiratory illness (SARI) in Kenya between 5th May 2020 and 31st December 2022. We explore risk factors for poor outcomes among those admitted with SARI during the COVID-19 pandemic. Additionally, we describe the effectiveness of licensed COVID-19 vaccines against admission with COVID-19 as well as mortality due to SARI.

## Methods

### Study design

We conducted a prospective multi-hospital clinical surveillance between 5th May 2020 and 31st December 2022 in 16 public hospitals in Kenya (Supplementary Fig. 1). The study sites were level four and level five hospitals part of a clinical information network (CIN) established in 2013 [17]. They are spread across the country’s densely populated western, central and coastal regions [18].

The surveillance was aimed at monitoring the evolution of the pandemic through review of admissions and management of patients with SARI in the adult medical wards. Additionally, it allowed for the estimation of COVID-19 vaccine effectiveness using a test-negative case-control study approach. Data were collected during the course of routine delivery of care and we did not influence the clinical management of patients in the wards or provide any support for laboratory testing for SARS-CoV-2. All patients admitted to the adult medical wards of the participating hospitals were included in the surveillance. Individual consent for access to de-identified patient data was waived for this surveillance study. There were no inclusion and exclusion criteria for those included in the surveillance. However, the analyses reported in this paper were restricted to those aged 18 years and above as some of the participating hospitals routinely had children aged between 16 and 18 years admitted to their adult medical wards. This study was approved by the Scientific and Ethical Review Unit (SERU) of the Kenya Medical Research Institute (KEMRI).

### Study patients

Data from all adult patients (age ≥ 18 years) discharged from medical wards in the participating sites during the study period were included. The discharge date and diagnosis were used to define inpatient episodes to ensure consistency in populations [18]. We collected data on the demographic and clinical characteristics, COVID-19 vaccination status, discharge diagnoses and clinical outcome of all included patients. Those with SARI had additional data on their clinical symptoms, laboratory results and management collected.

Using Kenyan MOH guidelines, SARI was defined as the presence of fever or cough or shortness of breath that required hospitalisation, in the absence of an alternative diagnosis that fully explains the clinical presentation [19–21]. Confirmed COVID-19 cases were patients with SARI who had a positive real-time reverse transcription-polymerase chain reaction (RT-PCR) or antigen detection rapid diagnostic test (Ag-RDT) for severe acute respiratory syndrome coronavirus 2 (SARS-CoV-2) in a respiratory sample during or before admission [21].

Admission and discharge diagnoses were recorded based on the International Classification of Diseases, Tenth Revision, Clinical Modification (ICD-10-CM) codes.

### Data collection

The data collection and analysis procedures for the CIN sites are reported elsewhere [22]. In summary, trained clinical officers abstracted data from patient records to an electronic data capture tool at each site daily. De-identified data were then synchronized to KEMRI-Wellcome Trust Research Programme (KWTRP) servers where a central data team conducted regular data checks. Additional checks included cross-validation of monthly tallies with daily bed returns reported at the hospitals [18].

A dynamic structured data collection tool was adapted for use. This tool was updated regularly in-keeping with emerging knowledge and changes in global and national guidelines for COVID-19 patient management. Key changes included (a) recording of steroid use among patients admitted with SARI following the WHO recommendation of dexamethasone use in management of COVID-19 in June 2020 [20, 23]; (b) use of Ag-RDT results in addition to RT-PCR results to confirm positive SARS-CoV-2 cases [24] and, (c) recording of patient vaccination status following the commencement of the COVID-19 vaccination program in Kenya in March 2021 [21].

### Statistical analysis

#### Mortality among patients admitted to the adult medical wards

We described the characteristics of all adult patients included in this surveillance study. We determined factors associated with progression to inpatient mortality among all patients using a forward-built stepwise random effects logistic regression model that accounted for clustering by hospital. For these analyses, patients were grouped into three groups (1) non- SARI, (2) SARI with a positive SARS-CoV-2 test at admission and (3) SARI with a negative SARS-CoV-2 test at admission. The final model included age, sex and presence of a chronic comorbidity as risk factors for mortality.

#### Predictors of mortality among patients with SARI

We used a forward stepwise approach to build a random effects logistic regression model to determine the factors associated with progression to inpatient mortality among patients admitted with SARI. This random-effects model accounted for clustering by the included hospitals. These analyses were limited to patients who had complete data on all potential risk factors, including age, sex, presence of a chronic comorbidity, leucocyte count at admission, haemoglobin level at admission, temperature at admission, oxygen saturation at admission and SARS-CoV-2 infection test results. These variables were defined as confounders if the crude and adjusted odds ratio differed by > 10% and/or the stratum specific odds ratios differed by > 10%.

## COVID-19 vaccine effectiveness

We used a test-negative case control design to estimate COVID-19 vaccine effectiveness (VE). We restricted these analyses to patients admitted after 5th March 2021 when COVID-19 vaccines were available in Kenya.

Patients were considered vaccinated if they had received a COVID-19 vaccine at least 14 days before their admission. Patients were grouped as having a ‘confirmed’ or ‘probable’ vaccination status based on whether their vaccination histories were verifiable on the national registry. We used a random effects logistic regression model, accounting for clustering by hospital, to estimate COVID-19 VE against the following outcomes: (a) admission with SARS-CoV-2 positive SARI (i.e., COVID-19), and (b) progression to inpatient mortality among those admitted with SARI (disregarding SARS-CoV-2 infection status because of low SARS-CoV-2 testing at the hospitals).

For VE against admission with SARS-CoV-2 positive SARI, we considered as cases SARI patients who tested positive for SARS-CoV-2 at admission while those who tested negative were considered controls. For VE against progression to inpatient mortality among those admitted with SARI, we considered SARI patients who died during admission as cases and those discharged alive as controls. Estimates were adjusted by age, sex and presence of a chronic comorbidity. VE was calculated as 1 minus the adjusted odds ratio multiplied by 100.

We could not estimate VE by full/partial vaccination status, VE against the predominant variant and VE against mortality due to confirmed COVID-19 because of limited SARS-CoV-2 testing, incomplete vaccination records, difficulty confirming the vaccination status for some patients from the national vaccination registry and mixing of vaccine products.

### Secondary analyses

Additional analyses reported in supplementary data include (1) a Kaplan Meier plot of cumulative mortality among all included patients by SARI and SARS-CoV-2 infection status, (2) predictors of progression to inpatient mortality among patients admitted with SARI analysed by a cox regression model, (3) estimation of COVID-19 vaccine effectiveness against progression to inpatient mortality among patients admitted with SARI using hazard ratios (HR)(VE calculated as 1 minus adjusted HR multiplied by 100).

All data were analysed using Stata/IC™ 16.0 (Stata Corp, College Station, Texas, USA).

## Results

### General characteristics of study patients

We collected data from 52,636 patients admitted across the 16 hospitals. Of these, 17,950 (34.1%) were patients classified as having SARI. Of all SARI patients, 4,880(27.1%) had complete data for inclusion in the analysis for predictors of mortality among SARI patients. The median age of patients was 50 years (interquartile range (IQR) 35 to 67 years). Distribution of patients by sex was equal. The median length of stay in hospital was 5 days (interquartile range (IQR) 3 to 9 days). Pneumonia was the most common discharge diagnosis with heart failure, COVID-19 and pulmonary tuberculosis being the second, third and fourth, respectively (Supplementary Table 1). One or more comorbidities at admission was present in 16.5% (*n* = 8,673) of all patients. The three most common chronic comorbidities among all included participants were hypertension (7.1%, *n* = 3,752), Human Immunodeficiency Virus (HIV) infection (5.1%, *n* = 2,688) and diabetes mellitus (3.8%. *n* = 1,973).

The proportion of patients receiving specialised care as part of their management i.e., receiving ventilation, intravenous (IV) fluids and/or oxygen was highest between April and June 2021 (13.1%; *n* = 1,341) and lowest between October and December 2022 (2.8%; *n* = 284) (Supplementary Table 2).

COVID-19 vaccination histories were available for 22,918 (43.5%) patients eligible for vaccination. Of these, 4.0% (*n* = 919) had received at least one dose of a COVID-19 vaccine. A SARS-CoV-2 test sample was collected from 48.4% (*n* = 8,692) of all SARI patients and results were available for 97.5% (*n* = 8,471) of these (Table 1).

**Table 1:** Distribution of all patients included in the surveillance by key demographic and clinical parameters

Category^a^*n* (all patients, patients admitted with SARI)		All patients *n* (%)	^b^Patients admitted with SARI *n* (%)
Age			
(52,633; 17,949)	18–30 years	8,346 (15.9%)	2,004 (11.2%)
	31–40 years	9,269 (17.6%)	2,939 (16.4%)
	41–50 years	8,455 (16.1%)	2,791 (15.6%)
	51–60 years	7,780 (14.8%)	2,962 (16.5%)
	61 years and above	18,783 (35.7%)	7,253 (40.4%)
Sex			
(52,625; 17,944)	Female	26,412 (50.2%)	8,467 (47.2%)
	Male	26,213 (49.8%)	9,477 (52.8%)
Presence of chronic comorbidity			
(52,636; 17,950)	None	43,963 (83.5%)	9,363 (52.2%)
	One	7,040 (13.4%)	6,970 (38.8%)
	Two	1,510 (2.9%)	1,494 (8.3%)
	Three or more	123 (0.2%)	123 (0.7%)
Length of stay			
(52,538; 17,927)	Less than 5 days	22,613 (43.0%)	6,787 (37.9%)
	5–7 days	8,546 (16.3%)	2,970 (16.6%)
	1 week – 2 weeks	14,243 (27.2%)	5,363 (29.9%)
	More than 2 weeks	7,136 (13.5%)	2,809 (15.6%)
Discharge Outcome			
(52,580; 17,932)	Dead	8,845 (16.8%)	3,827 (21.3%)
	Alive	43,735 (83.2%)	14,105 (78.7%)
Temperature			
(^c^,15,668)	Hypothermia (< 36.0^o^C)	^c^	2,310 (14.7%)
	Normal	^c^	11,514 (73.5%)
	Hyperthermia (> 37.7 ^o^C)	^c^	1,844 (11.8%)
Oxygen Saturation			
(^c^, 16,659)	Less than 90%	^c^	6,592 (38.8%)
	90–94%	^c^	4,107 (24.7%)
	More than 95%	^c^	6,160 (36.5%)
White Blood Cell count			
(^c^, 10,961)	Leukopenia (< 4.5*10^9^/L)	^c^	1,572 (14.3%)
	Normal	^c^	6,551 (59.8%)
	Leukocytosis (> 12*10^9^/L)	^c^	2,838 (26.9%)
Hemoglobin levels			
(^c^, 13,822)	Anemia (< 11.5 g/dL)	^c^	5,465 (39.5%)
	Normal	^c^	8,357 (60.5%)
Malaria results			
(^c^,4,405)	Negative	^c^	3,958 (89.9%)
	Positive	^c^	447 (10.1%)
SARS-CoV-2 test results			
(^c^, 8,460)	Negative	^c^	6,096 (72.1%)
	Positive	^c^	2,364 (27.9%)

^a^n for each parameter varies due to variable documentation of these data and/or variable testing for these parameters

^b^Patients admitted with SARI are a subgroup of all included patients

^c^Data not collected

Overall, 16.8% (*n* = 8,845/52,580) of the included patients died in hospital (Table 1). Of the 3,827 (21.3%) SARI patients who died in hospital, 531 (13.9%) were confirmed to be positive for SARS-CoV-2 at admission. The rest were negative for SARS-CoV-2 (31.3%, *n* = 1,196) or were not tested for SARS-CoV-2 at admission (54.9%, *n* = 2,100).

### Inpatient mortality among patients admitted to the adult medical wards

Of the patients with data on their discharge outcome, case fatality among patients without SARI (non-SARI patients) was 14.5% (5,018 out of the 34,648 patients who didn’t have SARI at admission), that of SARI patients with a positive SARS-CoV-2 test at admission was 22.5% (531 out of 2,363 SARI patients who were positive for SARS-CoV-2 while that of SARI patients with a negative SARS-CoV-2 test was 21.2% (3,296 out of the 15,615 SARI patients who had a negative SARS-CoV-2 test. Using non-SARI patients as the reference category, the crude odds ratio (OR) for mortality was 1.52 (95% confidence interval (CI) 1.45–1.61; *p* value < 0.0001) for SARI patients who were negative for SARS-CoV-2 and 1.47 (95% CI 1.33–1.64; *p* value < 0.0001) for SARI patients who tested positive for SARS-CoV-2 at admission. The difference was reduced after adjusting for age, sex and presence of a chronic comorbidity (SARS-CoV-2 negative SARI aOR 1.22, 95% CI 1.10–1.37; SARS-CoV-2 positive SARI aOR 1.32, 95% CI 1.24–1.40; *p*-value < 0.0001). In these analyses, male sex, presence of a chronic comorbidity and increasing age were associated with a higher rate of mortality. Supplementary Fig. 2 displays cumulative in-patient mortality for SARI and non-SARI patients.

Table 2 summarizes the predictors of mortality among patients admitted with SARI after adjusting for infection with SARS-CoV-2, age group, presence of a chronic comorbidity, temperature, oxygen saturation, white blood cell counts and haemoglobin levels at admission. Overall, male sex, oxygen saturation of less than 90%, anaemia, hypothermia, leucocytosis, presence of a chronic comorbidity and having a positive SARS-CoV-2 test at admission were the strongest predictors of mortality among these patients.

**Table 2:** Multivariable logistic regression analyses on predictors of progression to inpatient mortality among SARI patients

Demographic or clinical parameter		Crude Odds ratio	95% CI	Adjusted Odds ratio	95% CI	Likelihood ratio test *p* value
SARS-CoV-2 infection status	Negative					0.0076
	Positive	**1.22**	**1.03**,** 1.46**	**1.28**	**1.07**,** 1.52**	
Age	18–30 years					0.0073
	31–40 years	1.07	0.78, 1.46	1.01	0.73, 1.40	
	41–50 years	1.14	0.84, 1.56	1.05	0.77, 1.45	
	51–60 years	0.99	0.72, 1.35	0.90	0.65, 1.24	
	61 years and above	**1.39**	**1.06**,** 1.83**	1.30	0.98, 1.74	
Sex	Female					< 0.0001
	Male	**1.49**	**1.28**,** 1.73**	**1.57**	**1.35**,** 1.82**	
Presence of a chronic comorbidity	None					0.0273
	At least one	1.14	0.99, 1.32	**1.16**	**1.00**,** 1.35**	
Temperature	Normal					0.0196
	Hypothermia (< 36.0^o^C)	**1.35**	**1.11**,** 1.66**	**1.39**	**1.13**,** 1.70**	
	Hyperthermia (> 37.7 ^o^C)	1.14	0.90, 1.44	1.11	0.88, 1.42	
Oxygen Saturation	More than 95%					< 0.0001
	90–94%	1.05	0.84, 1.33	1.06	0.84, 1.34	
	Less than 90%	**1.72**	**1.41**,** 2.08**	**1.69**	**1.38**,** 2.06**	
White Blood Cell count	Normal					< 0.0001
	Leukopenia (< 4.5)	1.16	0.92, 1.45	1.15	0.92, 1.45	
	Leukocytosis (> 12)	**1.54**	**1.31**,** 1.81**	**1.54**	**1.30**,** 1.81**	
Hemoglobin levels	Normal					< 0.0001
	Anemia (< 11.5)	**1.34**	**1.15**,** 1.56**	**1.50**	**1.28**,** 1.76**	

### COVID-19 vaccine effectiveness

Of all vaccinated patients included in the VE analysis (*n* = 356), 20.5% (*n* = 73) had received the AstraZeneca, 8.4%(*n* = 30) had received the Janssen, 3.4%(*n* = 12) had received the Moderna and 7.6%(*n* = 27) had received the Pfizer vaccine product as their first dose. The rest, 60% (*n* = 214) reported being vaccinated but did not have a vaccine certificate to verify the vaccine product they had received. Sensitivity analysis revealed no difference between probable and confirmed vaccinees by age, sex, presence of a chronic comorbidity and SARS-CoV-2 test results. For SARI patients with vaccination histories and SARS-CoV-2 results (*n* = 3834, Table 3), 19.1% (4/22), 20.0% (1/8) and 24.5% (164/785) of all deaths among the probable vaccinees, confirmed vaccinees and unvaccinated patients respectively were among those with positive SARS-CoV-2 tests.

**Table 3:** COVID-19 vaccine effectiveness

Vaccine effectiveness against progression to inpatient mortality among patients admitted with SARI						
1. **Vaccination status**						
	**Alive (controls)**	**Dead (cases)**	**Crude VE**	**95% CI**	**Adjusted VE**	**95% CI**
Unvaccinated	5852	1417	-	-	-	-
Probable vaccination^a^	177	37	0.17	-0.19, 0.42	0.20	-0.15, 0.45
Confirmed vaccination	129	13	**0.59**	**0.27**,** 0.77**	**0.59**	**0.27**,** 0.77**
2. **Vaccine Product (first dose)**						
	**Alive (controls)**	**Dead (cases)**	**Crude VE**	**95% CI**	**Adjusted VE**	**95% CI**
Unvaccinated	5852	1417	-	-	-	-
AstraZeneca	67	6	**0.62**	**0.13**,** 0.84**	**0.64**	**0.14**,** 0.84**
Jannsen (Johnson & Johnson)	27	3	0.55	-1.51, 0.86	0.53	-0.50, 0.87
Moderna	11	1	0.64	-1.83, 0.95	0.63	-1.90, 0.95
Pfizer	24	3	0.51	-0.63, 0.85	0.46	-0.70, 0.85
**Vaccine effectiveness against admission with SARS-CoV-2 positive SARI**						
1. **Vaccination status**						
	**Negative (controls)**	**Positive (cases)**	**Crude VE**	**95% CI**	**Adjusted VE**	**95% CI**
Unvaccinated	2863	785	-	-	-	-
Probable vaccination^a, b^	94	22	0.05	-0.60, 0.44	0.05	-0.61, 0.43
Confirmed vaccination^b^	62	8	**0.35**	**0.41**,** 0.71**	0.38	-0.39, 0.72
2. **Vaccine Product (first dose)**						
	**Negative (controls)**	**Positive (cases)**	**Crude VE**	**95% CI**	**Adjusted VE**	**95% CI**
Unvaccinated	2863	785	-	-	-	-
AstraZeneca	37	5	0.18	-1.16, 0.69	0.22	-1.09, 0.71
Jannsen (Johnson & Johnson)	8	1	0.18	-5.69, 0.90	0.02	-7.11, 0.88
Moderna	8	1	0.54	-3.14,0.95	0.59	-2.73, 0.96
Pfizer	9	1	0.71	-1.62, 0.97	0.71	-1.62, 0.97

^a^These patients reported being vaccinated and had text message confirmation of the vaccination (including the date of vaccination), but their vaccination records were not verifiable on the national COVID-19 vaccine registry

^b^Number of vaccinated patients included reduced because of missing SARS-CoV-2 test results for some vaccinated patients

COVID-19 vaccine effectiveness (VE) against progression to inpatient mortality following admission with SARI for those with a confirmed vaccination status after adjusting for age, sex and presence of a chronic comorbidity was 0.59, (95% CI 0.27–0.77, Table 3). This adjusted VE (aVE) was highest for those confirmed to have received the AstraZeneca vaccine product 0.63 (95% CI 0.14–0.84) compared to the other vaccine products (Table 3). Adjusted VE against admission to hospital with SARS-CoV-2 positive SARI for those with a confirmed vaccination status was not statistically significant (aVE 0.38, 95% CI -0.39–0.72, Table 3).

## Discussion

The value of surveillance systems during the COVID-19 pandemic cannot be overstated [9]. Insights from these systems and other hospital-based studies have been useful in identifying those at increased risk for severe outcomes and informing both pharmaceutical and non-pharmaceutical interventions against the disease [6, 25–29]. Our study found that patients admitted between May 2020 and December 2022 with SARI had a higher mortality rate compared to their non-SARI counterparts. Our study identified male sex, oxygen saturation of less than 90%, anaemia, hypothermia, leucocytosis, presence of a chronic comorbidity and having a positive SARS-CoV-2 test at admission as risk factors for death in patients admitted with SARI during the COVID-19 pandemic in Kenya.

While these findings cannot be directly compared to those from studies evaluating risk factors for death among patients admitted with confirmed COVID-19, predictors of mortality identified by our study are similar to those identified by these studies e.g., being advanced in age [5, 7, 10, 30–34], being male [3, 5–7, 10, 32, 33], having leukocytosis [30, 32, 35, 36], or a low oxygen saturation [34].

The increased mortality among patients admitted with SARI included in our analyses may be explained by disruptions in access to, availability of care and service delivery within the larger health system in Kenya during the pandemic that have been described by Barasa et al. [37]. These disruptions included inadequate access to specialized care like invasive ventilation and, disruptions in the supply of medical commodities that may have been useful for patients who were at an increased risk for poor outcomes during the COVID-19 pandemic [37]. Additionally, there was limited or variable testing for co-infection with other infectious diseases like malaria and HIV which are endemic to our region and are known to increase the risk of mortality among patients with SARI [38]. For example, routine testing for malaria among patients included in this surveillance was low with only 24.9% of adults tested. Unfortunately, routine testing for malaria was one of the services disrupted by the pandemic in Kenya and may have caused some of the patients with co-infection to miss out on treatment for the same [39]. While we report a reduction in hospitalisations for adults and in-hospital mortality during the COVID-19 period compared to the pre-COVID-19 period elsewhere [18], our study still observed the disproportionate burden of mortality due to SARI with positive SARS-CoV-2 infection among male patients and those with a chronic comorbidity compared to female patients and those without chronic comorbidities as has been reported globally.

Estimating vaccine effectiveness using observational studies, especially those in low- and middle-income settings such as ours, is challenging and not without shortcomings [40]. We report a modest adjusted COVID-19 vaccine effectiveness of 59% against progression to inpatient mortality among patients admitted with SARI, regardless of their SARS-CoV-2 infection status, during the COVID-19 pandemic in Kenya. This adjusted VE appeared to be higher among patients who received the AstraZeneca vaccine product at 64%, but a formal comparison between the different products was limited by small numbers of patients that received the other brands. Additionally, we report an adjusted COVID-19 vaccine effectiveness of 38% against admission with SARI with positive SARS-CoV-2 infection during the same period. However, the analyses for this aVE were limited by the low number of eligible patients due to low testing for SARS-CoV-2 at the study sites and a low proportion of vaccinated patients being included in the surveillance. Among the patients included in this surveillance study, only 48.4% of those with SARI were tested for COVID-19 and only 4.6% (*n* = 356) of those with SARI and eligible for vaccination had received at least one dose of a COVID-19 vaccine. Consequently, none of the reported results were statistically significant. In comparison to VE estimates reported globally and in sub-Saharan Africa, our reported aVE of 38% against admission with SARS-CoV-2 positive SARI (i.e., COVID-19) is lower than the 33–56% reported in the UK [41], 42–50% in Denmark [42], 65% in USA [43], 50–63% in Brazil [44], 53–71% in the UK [45], 58–71% in USA [46] and 70% in South Africa [47]. However, these studies reported aVE following a completed primary series of immunization while our study reports aVE following receipt of at least one dose of a COVID-19 vaccine. Our reported aVE of 59% against progression to inpatient mortality is slightly lower than that reported in Zambia for a similarly designed study. In this study, Chanda et al. report an aVE of 65% for patients who had equally received at least one dose of a COVID-19 vaccine against progression to inpatient mortality [48]. Nevertheless, our reported aVE of 64% for the AstraZeneca vaccine product against progression to inpatient mortality among patients admitted with SARI is similar to that reported for the same vaccine product in Zambia by Chanda et al.., 65% [48] and in India, 67% [49]. Our estimates, while informative, should be interpreted with caution as they are reported against a backdrop of extensive community spread of SARS-CoV-2 before vaccine introduction [50], limited access to and availability of SARS-CoV-2 test-kits and low vaccine coverage with variable availability of the vaccine [14]. Difficulty in data linkage across national, regional and local SARS-CoV-2 infection testing, vaccination registries and hospital admissions limited the patients included in these analyses. Consequently, the estimates reported are not without bias [51]. Specifically, the effect of selection bias, healthy vaccinee bias, misclassification, waning immunity and differential depletion of susceptibles on the final estimates cannot be overlooked. Additionally, limited capacity to verify verbal vaccination histories and mixing of vaccine products due to variable availability of specific vaccine products hindered our study’s ability to estimate VE by all licensed vaccine products.

### Strengths and limitations

A major strength of our study is the large sample size achieved during the study period. Where data on clinical outcomes in patients with COVID-19 in our region are limited [52], these results provide key insights on the pandemic within our setting. Our study developed a dynamic data collection tool that allowed real-time review of management practices and clinical outcomes with evolving knowledge on the disease. Whilst the study was not designed to evaluate emerging treatment modalities for COVID-19, its findings on the use of steroids in treating SARI have allowed for further studies to be conducted [53].

Despite including multiple sites spread across densely populated areas of the country and having a large sample, the results of this study may not be fully generalizable to the country. Our sample of patients testing positive for SARS-CoV-2 infection represents only about 1% of all cases reported in the country. However, the results are still relevant both nationally and globally.

Relying on routine clinical data and documentation practices which differ by individual and by site limited the study’s ability to explore the effect of other known risk factors for death in COVID-19 such as multiple comorbidities at admission, social habits e.g., smoking, HIV infection or exposure to previous medication [6, 10, 29]. While completion of demographic parameters was high, poor documentation practices and missing data for clinical parameters and investigations limited the number of patients included in the multivariable analysis. This variability in documentation may have introduced bias to our estimates while limiting our capacity to describe the changing clinical pattern of those with COVID-19 by dominant SARS-CoV-2 variant or to explore the outcome in patients admitted with COVID-19 by evolving treatment practices. Finally, the effect of other confounding factors such as socioeconomic status, distance to the nearest health facility or COVID-19 vaccine hesitancy that were not evaluated by this study on the outcome and effects reported by this study cannot be overlooked. Future studies could aim to explore the effect of these factors on inpatient mortality and vaccination with COVID-19 vaccines.

Despite these limitations, the study identifies at-risk populations and highlights the need for improved documentation practices, access to specialized care and medical supplies, and more comprehensive testing for co-infections to improve the outcomes of COVID-19 patients in Kenya.

### Future of study

The COVID-19 pandemic has highlighted the need for effective hospital surveillance systems in Kenya [11]. By monitoring disease activity within the population in real-time, using signs and symptoms, or preliminary diagnoses, hospital surveillance systems can indicate changes in disease trends that require immediate attention [54]. Standardization of the minimum clinical data documented at health facilities is necessary to effectively monitor disease activity. Developing a standardized admission tool or collecting key priority information from hospitals and transmitting these data to a national repository would enable data linkage and analysis, thus improving surveillance and planning. Additionally, expanding our study to include real-time on-demand analysis [10] could further enhance the effectiveness of the hospital surveillance system.

## Conclusion

Our study has generated valuable clinical data on mortality among patients admitted to adult medical wards in the country and estimates of COVID-19 vaccine effectiveness during the COVID-19 pandemic. These data were useful in providing situational awareness during the first three years of the pandemic in Kenya and informing the national response measures. We also demonstrate the potential for routine clinical surveillance systems to inform public health activities in low resource settings.

## Supplementary Information

Supplementary Material 1.

## Data Availability

The dataset supporting the conclusions of this article is available in the Harvard Dataverse repository, https://doi.org/10.7910/DVN/1QXHPK.
