# Supplementary material for: Prospective clinical surveillance for severe acute respiratory illness and COVID-19 vaccine effectiveness in Kenyan hospitals during the COVID-19 pandemic
Source: BMC Infect Dis. 2024 Nov 5;24:1246. doi: 10.1186/s12879-024-10140-6 (PMC11536953; doi:10.1186/s12879-024-10140-6)
Supplement: Supplementary file 1 — Supplementary Material 1. [file 12879_2024_10140_MOESM1_ESM.pdf]

Prospective Clinical Surveillance for Severe Acute Respiratory Illness and COVID-19 Vaccine Effectiveness in Kenyan Hospitals during the COVID-19 pandemic - Supplementary Figures and Tables

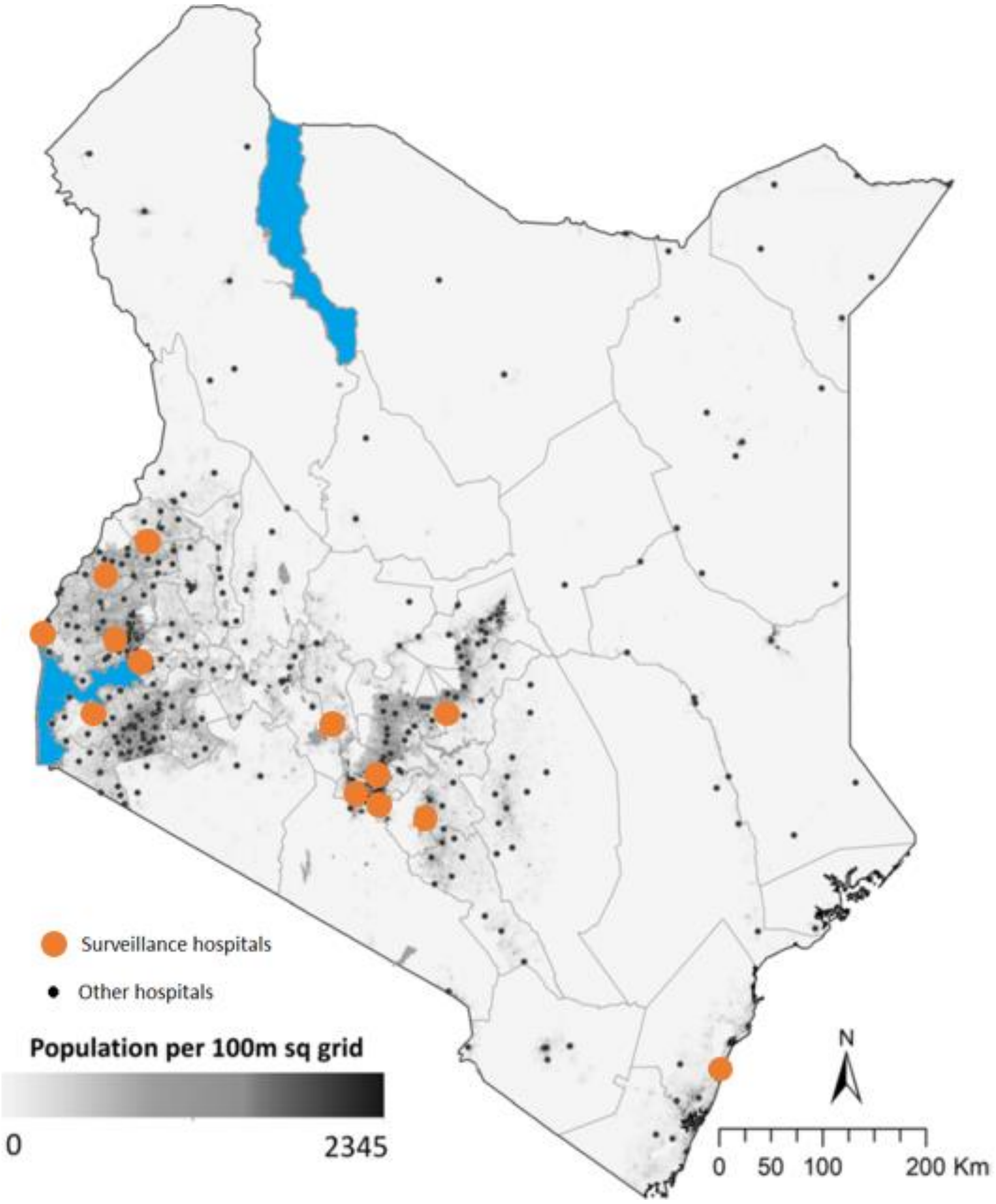

Supplementary Figure 1: Distribution of hospitals participating in the surveillance study

Supplementary Table 1: Distribution of discharge diagnoses for included patients overall and by their severe acute respiratory illness (SARI) status at admission

***Discharge diagnosis**	**Overall** n (% of all admissions)	**SARI** n (% of all SARI patients)	**Non-SARI** n (% of all non-SARI patients)
Pneumonia	3,285 (6.4 %)	2,788 (15.8 %)	497 (1.5 %)
Heart failure	2,639 (5.1 %)	1,334 (7.6 %)	1,305 (3.9 %)
COVID-19	1,896 (3.7 %)	1,827 (10.4 %)	69 (0.2 %)
Tuberculosis	1,852 (3.6 %)	1,449 (8.2 %)	403 (1.2 %)
Malaria	1,670 (3.2 %)	331 (1.9 %)	1,339 (4.0 %)
Gastroenteritis	1,596 (3.1 %)	190 (1.1 %)	1, 406 (4.1 %)
Sepsis	1,114 (2.2 %)	208 (1.2 %)	906 (2.7 %)
Meningitis	1,032 (2.0 %)	166 (0.9 %)	866 (2.6 %)
Chronic neurological disease	181 (0.4 %)	20 (0.1 %)	161 (0.5 %)
Other[a]	36,324 (70.4 %)	9,344 (52.9 %)	26,980 (79.5 %)
Total[b]	51,589 (100 %)	17,657 (100.0 %)	33,932 (100.0 %)

***** Discharge diagnoses represent the actual medical condition for which a patient was treated while admitted

[a] Those with a discharge diagnosis of ‘other’ were patients who had other diagnoses that are expected among patients admitted to adult medical wards, but these were too few to be grouped into categories of their own.

[b] Overall, 293 SARI patients and 754 non-SARI patients did not have their discharge diagnosis recorded

Supplementary Table 2: Distribution of patients admitted with severe acute respiratory illness (SARI) who died or received specialised care for by year of surveillance

Year	Quarter of Year	[a] Patient Characteristics		
		% Deaths (n = 8,845)	% Managed in Isolation (n = 2,791)	% Required Specialised Care (n = 10,228)
**2020**	*May to June 2020	7.1 (627)	4.3 (123)	7.4 (756)
	July to September 2020	11.9 (1,051)	15.6 (436)	12.1 (1,238)
	October to December 2020	8.5 (752)	20.1 (557)	10.5 (1,074)
**2021**	January to March 2021	8.9 (788)	12.7 (354)	9.3 (953)
	April to June 2021	12.6 (1,114)	18.9 (526)	13.1 (1,341)
	July to September 2021	13.3 (1,176)	18.5 (515)	9.7 (991)
	October to December 2021	7.0 (615)	4.8 (133)	5.4 (547)
**2022**	January to March 2022	8.0 (704)	3.8 (108)	11.1 (1,135)
	April to June 2022	9.7 (861)	0.07 (2)	9.2 (943)
	July to September 2022	10.2 (906)	0.7 (20)	9.4 (966)
	October to December 2022	2.8 (251)	0.6 (17)	2.8 (284)

[a]Chi square test of difference in distribution of patients by quarter across the three patient characteristics groups had a p value < 0.0001

*Data for April 2020 not included in these analy**ses** as the surveillance had not begun. Surveillance began on 5th May 2020

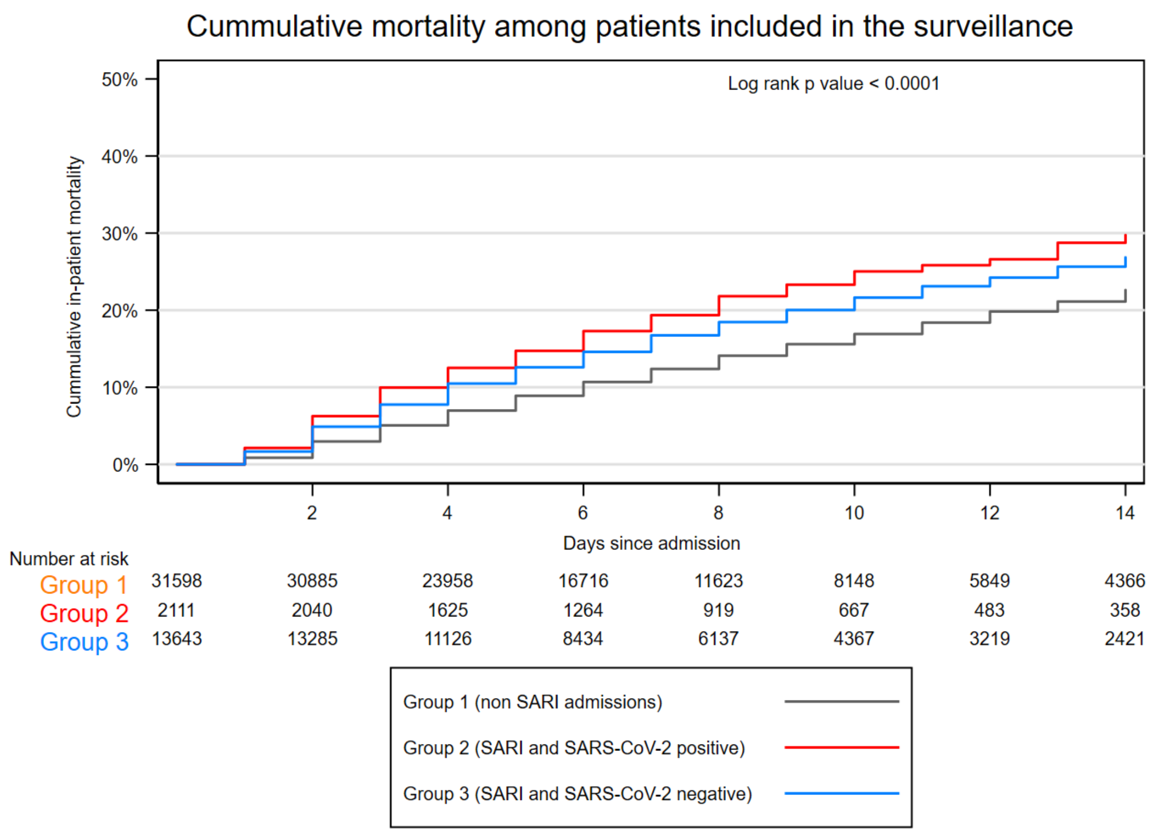

Supplementary Figure 2: Kaplan Meier plots of cumulative in-patient mortality among all included patients overall and by their SARI status

Supplementary Table 3: Multivariable cox regression analysis of the predictors of progression to inpatient mortality among patients admitted with SARI

		**Crude rate ratio**	**95% CI**	**Adjusted rate ratio**	**95% CI**	***LRT p value**
COVID status	Negative					0.0012
	Positive	**1.30**	**1.12, 1.53**	**1.31**	**1.12, 1.54**	
Age	18 – 30 years					0.0049
	31 – 40 years	1.12	0.81, 1.53	1.05	0.77, 1.44	
	41 – 50 years	1.02	0.74, 1.40	0.92	0.67, 1.27	
	51 – 60 years	1.00	0.73, 1.37	0.90	0.65, 1.22	
	61 years and above	**1.38**	**1.05, 1.81**	1.25	0.95, 1.66	
Sex	Female					0.0001
	Male	**1.35**	**1.16, 1.56**	**1.37**	**1.18, 1.59**	
Presence of a chronic comorbidity	None					0.4891
	At least one	1.02	0.88, 1.17	1.05	0.91, 1.22	
Temperature	Normal					0.0766
	Hypothermia (< 36.0ºC)	1.20	1.00, 1.46	**1.23**	**1.02, 1.49**	
	Hyperthermia (> 37.7 ºC)	1.08	086, 1.35	1.11	0.89, 1.39	
Oxygen Saturation	More than 95%					< 0.0001
	90% to 94%	**1.08**	**0.86, 1.35**	**1.08**	**0.86, 1.35**	
	Less than 90%	**1.57**	**1.33, 1.87**	**1.55**	**1.30, 1.86**	
White Blood Cell count	Normal					0.0002
	Leucopenia (< 4.5)	1.06	0.85, 1.33	1.11	0.90, 1.39	
	Leukocytosis (> 12)	**1.37**	**1.17, 1.61**	**1.38**	**1.18, 1.62**	
Hemoglobin levels	Normal					< 0.0001
	Anemia (< 11.5)	1.08	0.94, 1.25	**1.26**	**1.08, 1.47**	

*LRT- Likelihood ratio test
[a]Cox regression model doesn't account for clustering by study sites

Supplementary Table 4: COVID-19 Vaccine Effectiveness based on multivariable cox regression analysis

**Vaccine effectiveness against progression to inpatient mortality among patients admitted with SARI**						
	Alive (controls)	Dead (cases)	Crude VE %	95% C. I	Adjusted VE %	95 % C. I
Unvaccinated	5852	1417	-	-	-	-
Probable vaccination [a]	177	37	0.25	-0.22, 0.54	0.26	-0.21, 0.55
Confirmed vaccination	129	13	**0.55**	**0.6, 0.79**	**0.53**	**0.4, 0.78**

[a] These patients reported being vaccinated with text message confirmation (including the date of vaccination) but their vaccination records were not verifiable on the national vaccine registry

[*] Cox regression model doesn’t account for clustering by study sites
